# Current Trends in Duchenne Muscular Dystrophy Research and Therapy: 3D Cardiac Modelling

**DOI:** 10.1002/jcsm.70180

**Published:** 2026-01-07

**Authors:** Marta Przymuszała, Marta Białobrzeska, Józef Dulak, Urszula Florczyk‐Soluch

**Affiliations:** ^1^ Department of Medical Biotechnology, Faculty of Biochemistry, Biophysics and Biotechnology Jagiellonian University Kraków Poland; ^2^ Doctoral School of Exact and Natural Sciences Jagiellonian University Krakow Poland

**Keywords:** 3D cardiac models, DMD therapy, Duchenne muscular dystrophy, gene therapy, hiPSCs

## Abstract

Duchenne muscular dystrophy (DMD), caused by dystrophin deficiency, presents a multifaceted challenge that affects both skeletal muscle function and cardiomyocyte homeostasis, causing progressive degeneration and life‐threatening cardiac complications by adolescence. Current treatments fail to prevent poor prognoses, and while FDA‐approved therapies show promise in targeting dystrophin restoration, including RNA‐based approaches and microdystrophin gene therapy, clinical evidence supporting their efficacy remains limited. Substantial challenges persist, particularly in achieving effective cardiac targeting, ensuring long‐term safety and developing scalable treatments. Alternative therapies addressing muscle and cardiac pathophysiology are being explored alongside dystrophin‐based approaches. DMD treatment is increasingly focusing on heart targeting with optimized cardiac‐specific delivery strategies. Human‐induced pluripotent stem cells (hiPSCs) enable DMD modelling, bridging pathophysiology and clinical phenotypes. DMD patient–specific hiPSC‐derived cardiomyocytes (hiPSC‐CMs) serve as in vitro models for disease mechanisms and therapy, with 3D cardiac models, either self‐organizing (spheroids) or moulded, expanding on hiPSC‐CMs to reflect cell interactions and myocardial tissue architecture. Advanced methods like 2D cell sheets, patches and engineered 3D human cardiac models show potential for improving cell engraftment and functional recovery in injured hearts, but their direct therapeutic application in DMD remains speculative due to extensive muscle mass loss; the complexity of cardiac and skeletal muscle interactions; and unresolved challenges related to cell integration, maturation and long‐term function. Considering the premature state of cell‐based therapies in this complex disease, current DMD treatment efforts focus on genetic approaches. Progress will likely depend on combining dystrophin‐restoring strategies with therapies targeting disease mechanisms and improving cardiac delivery.

## 1 Setting the Stage: Challenges in Duchenne Muscular Dystrophy (DMD)

The adult human heart exhibits impaired cardiomyocyte renewal and limited repair mechanisms [1]. Under normal myocardial homeostasis, pre‐existing cardiomyocytes serve as the primary source of cardiomyocyte replacement, yet their regenerative response to injury remains insufficient [2]. DMD poses a complex pathological challenge to cardiomyocyte homeostasis. The loss of functional dystrophin, a key component of the transmembrane dystrophin‐associated glycoprotein complex (DAGC), destabilizes the sarcolemma and disrupts its interaction with the extracellular matrix (ECM), leading to progressive degeneration and a broad range of consequences on muscle cell function [3]. This and the heart's limited capacity for regeneration severely impair the ability to repair cardiac tissue, resulting in structural and functional abnormalities that ultimately contribute to heart failure.

*DMD* mutations on the X chromosome are associated with the absence or insufficiency of functional dystrophin and are linked with a pooled global birth prevalence of approximately 1 in 5000 live male births [4]. DMD typically develops in early childhood with skeletal muscle symptoms, leading to the loss of walking ability around age 12, followed by respiratory muscle weakness. In parallel, silent cardiac disease begins by age 6 and progresses into clinical stages, such as conduction defects, dilated cardiomyopathy (DCM) and hypertrophic cardiomyopathy, with an onset usually occurring after 10 years of age and becoming evident in all patients by age 18 [5]. Based on systematic reviews of individual patient datasets (2283 patients, 14 publications [6] and 2662 patients, 15 publications [7]), current treatment standards, including ventilatory support, have prolonged the median survival of DMD patients to over 30 years, with an estimate of life expectancy of 28.1 (25.1–30.3) [6] and 29.9 years (26.5–30.8) [7] in respective analyses.

Despite therapeutic advances, cardiomyopathy remains the leading cause of death in DMD. Current strategies, such as early detection, medication and skeletal muscle therapies, improve the quality of life but fail to prevent cardiac decline. Tools like cardiac MRI and biomarkers (e.g., troponins and NT‐proBNP) are essential for monitoring heart damage and guiding timely interventions. Longitudinal clinical data show that left ventricular (LV) function in DMD declines despite current treatments, with considerable variability in the rate and age of onset. Multivariable analyses show that elevated B‐type natriuretic peptide (BNP) levels and the need for diuretics are stronger independent predictors of adverse outcomes than reduced LV ejection fraction (LVEF) alone, even in patients receiving ventilatory support [8].

Due to unmet needs in DMD, genetic therapies aim to restore dystrophin and improve muscle function. Since 2016, five have FDA approval: four RNA‐based and one adeno‐associated vector (AAV)–based microdystrophin therapy. Questions regarding trial design and outcome measures lead to doubts about the rigour of the current evidence to support informed prescribing [9]. Although ambulatory outcomes show benefits, none of the marketed therapies demonstrate uptake or activity in cardiac tissue, a critical concern for full DMD treatment. This creates a need for in vitro personalized cardio‐specific models to test novel therapeutic strategies.

Direct therapeutic targeting of dystrophin deficiency is challenged by the need to target large muscle mass, the extreme size of the *DMD* gene, 90 times larger than average and extensive genetic alterations, with a mutation rate exceeding that of other human genes (1.10^−4^ vs. 10^−5^–10^−6^) [10]. Ongoing efforts aim to restore dystrophin in skeletal and cardiac muscles and address the consequences of its deficiency. Combining approaches may be synergistic but needs further validation. A major challenge is identifying safe, effective therapies for patients, requiring advanced human models and thorough preclinical and genetic studies.

hiPSC‐CMs enable patient‐specific cardiac modelling of DMD in 2D cultures, linking molecular mechanisms with clinical phenotypes. Advanced 3D cardiac constructs better replicate myocardial architecture and function, offering more physiologically relevant platforms to study disease progression and test emerging therapies. Building on these models, ongoing research increasingly focuses on refining genetic interventions to improve cardiac outcomes in DMD.

## 2 Genetic Innovations in DMD: Therapeutics and Current Research

Genetic therapies in DMD aim to restore dystrophin expression in muscle cells, targeting the minimal level needed for functional benefit. In 3‐month‐old female heterozygous mice, 50% dystrophin expression in cardiomyocytes reduced β‐isoproterenol‐induced cardiomyopathy in *mdx* mice during in vivo hemodynamic tests [11]. Similarly, in 21‐month‐old carrier *mdx* mice, mosaic dystrophin or complementary utrophin expression protected against DCM. Under dobutamine stress, these mice maintained near‐normal haemodynamics with dystrophin present in 50% of cardiomyocytes and increased utrophin in dystrophin‐deficient cells [12]. These results suggest that 50% cardiac dystrophin expression may be a sufficient therapeutic target. However, the *mdx* model's limitations, its milder and delayed cardiomyopathy compared with human DMD, require surrogate endpoints, reducing the translational relevance of these results [13].

In humans, studies on X‐linked DCM, caused by *DMD* mutations with exclusive cardiac involvement, show that 29%–57% dystrophin expression can prevent muscle weakness, suggesting a threshold of ~30% is needed to avoid DMD development [14]. Complementary insights come from human female carriers of DMD mutations, whose mosaic dystrophin expression due to X‐chromosome inactivation provides a natural model to study how variable dystrophin expression affects cardiac function [15]. Though they may not show skeletal symptoms, carriers often develop myocardial fibrosis, reduced LVEF and conduction defects, mimicking full DMD cardiac pathology [16]. Cardiac MRI frequently reveals late gadolinium enhancement and LV hypertrabeculation, supporting the need for early and regular cardiac monitoring [17]. Symptoms can arise in childhood or adulthood and may worsen during stress events (e.g., pregnancy and anaesthesia). These findings emphasize the risks of incomplete dystrophin restoration, as residual dystrophin‐negative cells may drive electrical instability. While ~50% dystrophin expression may yield functional improvement, evidence from carriers shows that mosaic patterns can still lead to serious cardiac complications, underscoring the importance of therapies achieving broad dystrophin expression across cells.

**FIGURE 1:**
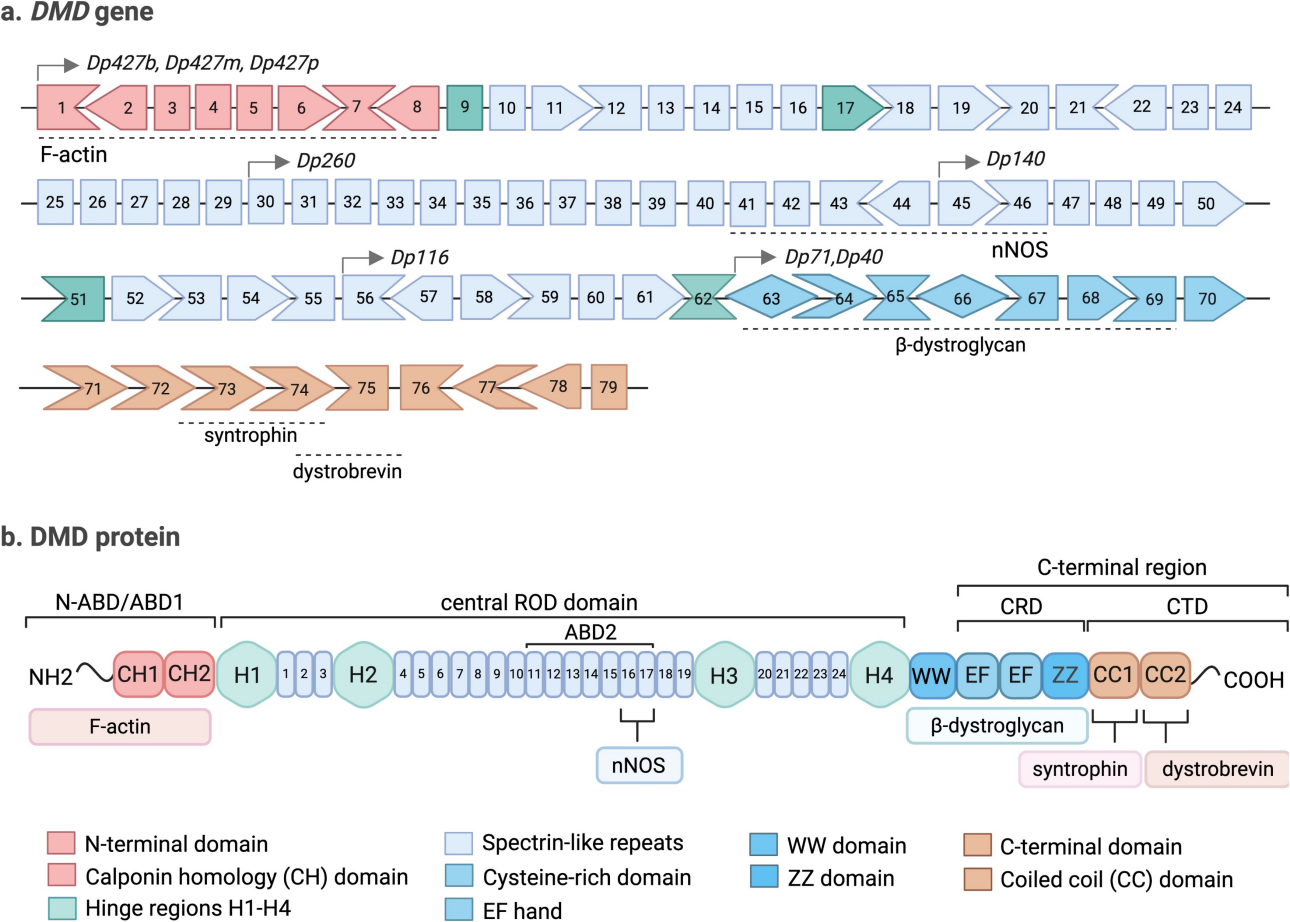
Schematic of DMD gene and dystrophin protein structure. (a) The *DMD* gene consists of 79 exons on the X chromosome (Xp21) and contains at least seven tissue‐specific promoters and two polyadenylation sites producing several dystrophin isoforms. Full‐length isoforms (Dp427b, Dp427m and Dp427p) are generated from three upstream promoters, while internal promoters drive shorter isoforms, including Dp260, Dp140, Dp116 and Dp71. The smallest isoform, Dp40, shares a first exon with Dp71 but uses a distinct polyadenylation site. (b) Full‐length dystrophin (427 kDa) is organized into four distinct domains. The N‐terminal actin‐binding sites (ABD1; encoded by Exons 1–8) binds F‐actin via two calponin‐homology (CH) domains. The rod domain (Exons 9–64) comprises 24 spectrin‐like repeats interspersed with four hinge regions (H), which provide flexibility and include ABD2 within Repeats 11–17. The cysteine‐rich domain (CRD; Exons 65–70) contains key motifs, including EF‐hand and ZZ, that mediate interactions with β‐dystroglycan. The C‐terminal domain (CT; Exons 71–79) features coiled‐coil regions that facilitate binding to syntrophin and dystrobrevin.

Building on these complexities, therapeutic targeting of dystrophin deficiency faces challenges, including the need to reach large muscle masses (both skeletal and cardiac), the extreme size of the DMD gene (2.3 Mb, 11 kb cDNA, 79 exons) (Figure 1) and extensive genetic variability. With thousands of mutations reported [18], the DMD gene has a mutation rate (1 × 10^−4^) much higher than the average human gene (10^−5^–10^−6^) [10]. Most mutations involve large intragenic rearrangements: 68%–70% deletions and 11% duplications, clustered in hotspot regions between Introns 43–55 (distal) and 1–20 (proximal). An additional 18%–20% are small mutations (mostly nonsense or frameshift) (based on combined analysis of 576 [19] and 7149 patients [18]).

**FIGURE 2:**
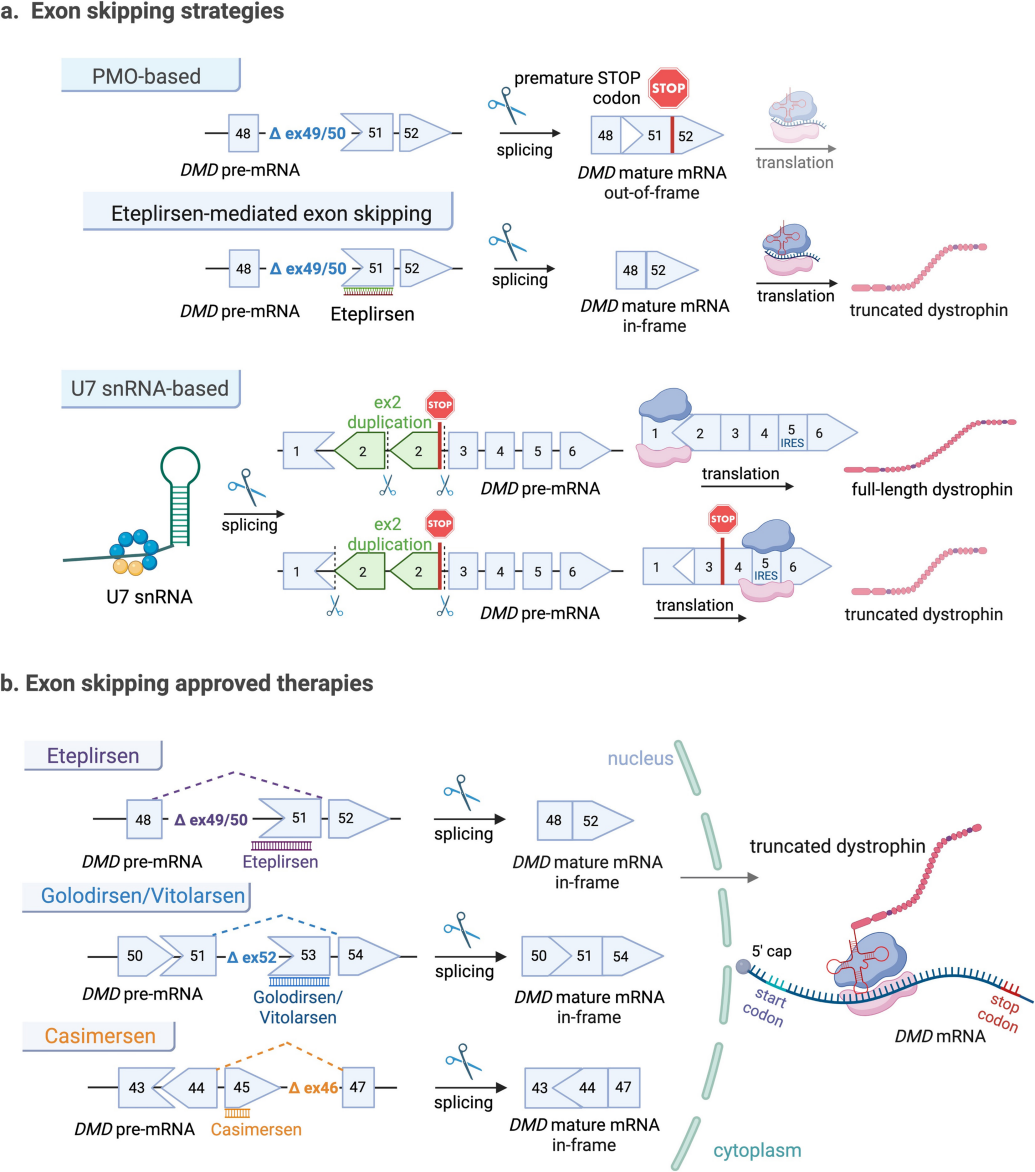
Therapeutic strategies based on targeting *DMD* mRNA processing. (a) Exon skipping. Phosphorodiamidate morpholino oligomers (PMOs), like eteplirsen, induce exon exclusion (e.g., Exon 51), restoring the reading frame and allowing functional dystrophin expression. The U7 snRNA‐based approach addresses splicing defects, such as Exon 2 duplication. Skipping one copy of Exon 2 restores the reading frame, producing full‐length dystrophin, while skipping both copies allows IRES in Exon 5 to facilitate translation, resulting in truncated dystrophin. (b) Approved PMO‐based treatments include eteplirsen (Exon 51), golodirsen and viltolarsen (Exon 53) and casimersen (Exon 45), each producing in‐frame dystrophin mRNA.

The identification of genetic mutations and hotspots has guided current dystrophin‐restoring strategies (Table S1), including mRNA‐based approaches such as stop codon read‐through and exon skipping. These strategies comprise stop codon read‐through, which bypasses premature stop codons, and exon skipping, which restores the reading frame, both allowing the production of shorter but functional dystrophin (Figure 2a). It is estimated that 58% of patients are eligible for single‐exon skipping, 63% for multiexon skipping (Exons 45–55) and 10%–14% for premature termination codon (PTC) read‐through therapies [18, 19].

### 2.1 DMD mRNA Modulation

Exon skipping is achieved using short, synthetic antisense oligonucleotides (ASOs), delivered systemically to bind specific exons during pre‐mRNA splicing, thereby blocking their inclusion in the mature transcript. Since 2016, four phosphorodiamidate morpholino oligomers (PMOs), a chemically modified type of ASOs, have received conditional FDA approval for DMD treatment: eteplirsen (Exondys 51) for Exon 51, golodirsen (Vyondys 53) for Exon 53, viltolarsen (Viltepso) for Exon 53 and casimersen (Amondys 45) for Exon 45 (Figure 2b and Table 1), with additional candidates in development [20].

**TABLE 1:** Approved genetic therapies in DMD.

Therapy type	Trade (generic) name	Chemistry	Mechanism	FDA approval	Negative vote from review or advisory committee	Broader age group in label compared with clinical trial	Age range of applicable patients	Route of administration	Dosing frequency
Exon skipping	Exondys 51 (eteplirsen)	PMO	Exon 51 skipping	Conditionally approved 2016	✓	✓	All	Intravenous	Weekly
	Vyondys 53 (golodirsen)	PMO	Exon 53 skipping	Conditionally approved 2019	—	✓	All	Intravenous	Weekly
	Viltepso (viltolarsen)	PMO	Exon 53 skipping	Conditionally approved 2020	—	✓	All	Intravenous	Weekly
	Amondys 45 (casimersen)	PMO	Exon 45 skipping	Conditionally approved 2021	—	✓	All	Intravenous	Weekly
Microdystrophin gene therapy	Elevidys (delandistrogene moxeparvovec)	AAV74	AAV74‐mediated microdystrophin therapy	Approval 2024	✓		Ambulatory 4 years and older	Intravenous	Single dose
				Accelerated approval 2024	✓		Nonambulatory 4 years and older	Intravenous	Single dose

Abbreviations: AAV—adeno‐associated virus, PMO—phosphorodiamidate morpholino oligomer.

The primary biological outcome, dystrophin expression in skeletal muscle biopsies (typically from the biceps brachii), is relatively comparable between drugs but varies across study cohorts. At Week 48, eteplirsen treatment increased the proportion of dystrophin‐positive fibres to approximately 52% in a small cohort (*n* = 4) [21], while in the larger Phase 3 PROMOVI trial (*n* = 32), levels reached around 16% [22]. Golodirsen reached 10% dystrophin‐positive fibres at the same timepoint [23].

While exon‐skipping therapies have shown slowed disease progression measured by functional tests (6‐min walk test and motor performance) and lung function (forced vital capacity) [22], ongoing safety monitoring remains critical due to reported adverse effects [24, 25]. Common issues include respiratory infections, hypersensitivity reactions (urticaria, rash and fever) and potential renal toxicity, with eteplirsen‐related events often emerging after prolonged treatment [25]. Long‐term use risks include liver accumulation, complement activation and thrombocytopenia [26]. Pharmacokinetic challenges further limit exon‐skipping effectiveness, with natural clearance reducing bioavailability of ASOs and their derivatives: PMOs and peptide‐conjugated PMOs (PPMOs). Moreover, poor cardiac delivery remains a critical hurdle [27]. To address this issue, PPMOs were developed with the goal of enhancing delivery to both skeletal and cardiac muscle. While PMO uptake was initially considered passive through leaky membranes, recent studies suggest it depends on the stage of myogenesis [27]. Peptide conjugation to the PMO backbone promotes endocytosis via scavenger receptor class A after self‐assembly into nanoparticles and improves pharmacokinetics. Larger clinical trials are crucial to evaluate their safety and efficacy, particularly for cardiac applications [24, 27].

**FIGURE 3:**
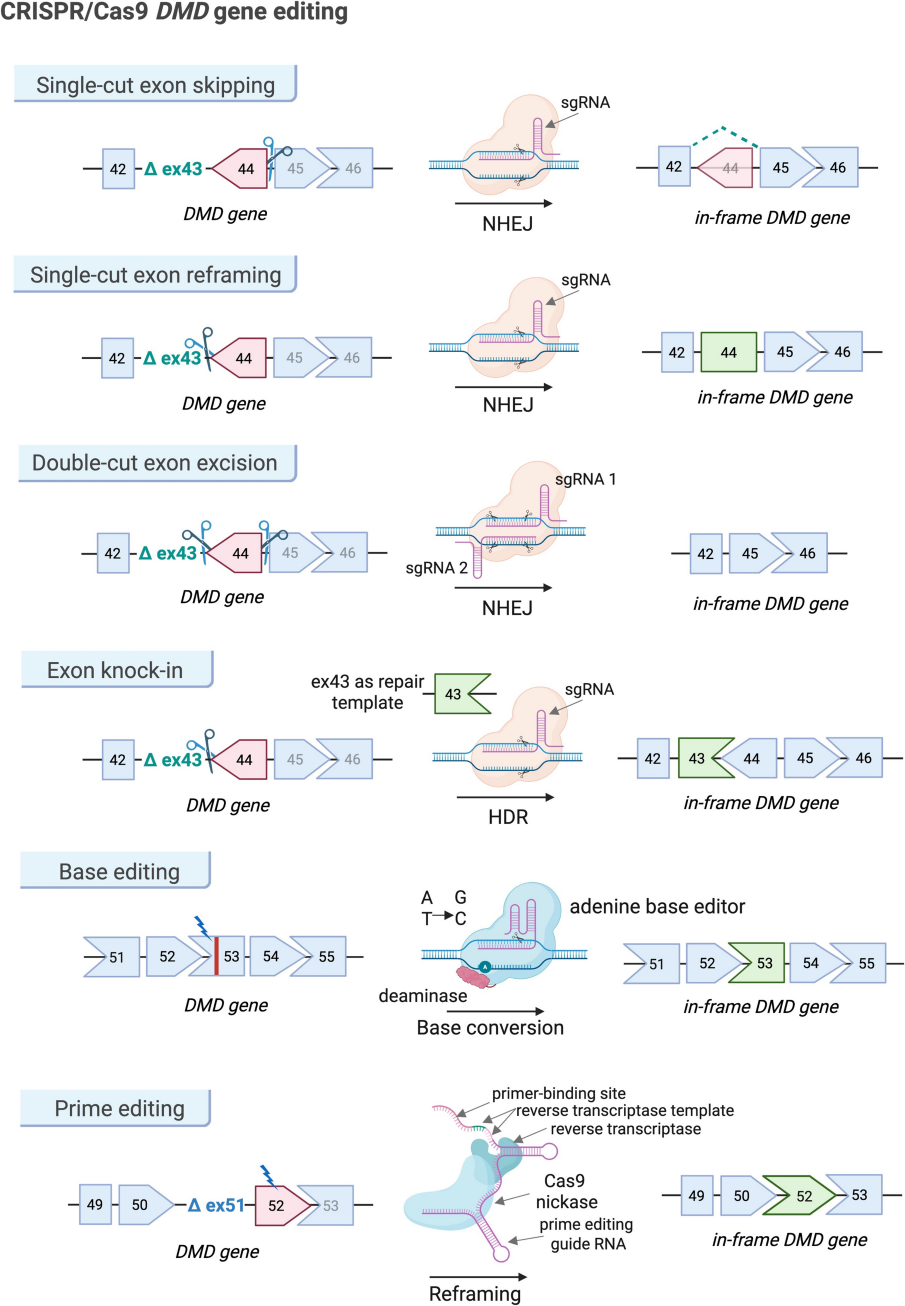
CRISPR/Cas9‐based gene‐editing strategies for restoring the *DMD* reading frame. Single‐cut exon skipping uses sgRNA to direct Cas9‐mediated exon skipping via NHEJ. Double‐cut exon excision removes the target exon with two Cas9 cuts. Single‐cut reframing modifies target exon by deleting or altering small sequences to restore the frame. Exon knock‐in uses HDR to correct mutant sequences. Prime editing uses a pegRNA that includes a primer‐binding site to hybridize with the target DNA strand, enabling the reverse transcriptase to copy the reverse transcriptase template into the genome. Base editing uses a Cas9 nickase and base‐modifying enzyme to precisely correct point mutations.

In DMD, PMOs and PPMOs represent the primary class of exon‐skipping drugs; however, additional mRNA‐level approaches are emerging from gene therapy (scAAV9.U7.ACCA), modified nucleic acid (ENA(2′‐O,4′‐C‐ethylene‐bridged nucleic acid)–based ASO) and RNase H‐mediated ASO technologies, all currently in Phase 1/2 clinical trials [24] (Figure 3).

scAAV9.U7.ACCA is an antisense therapy utilizing modified U7 small nuclear RNA (U7 snRNA) to induce exon skipping, specifically targeting Exon 2 duplications (Figure 2). Unlike traditional ASOs, it uses AAV Serotype 9 as a delivery vector. In non‐human primates, it showed efficient Exon 2 skipping in cardiac tissue with no reported adverse effects [28]. The therapy is currently in Phase 1/2 clinical trials for DMD patients with Exon 2 duplication.

Another mRNA‐targeting strategy is premature stop codon read‐through therapy, which addresses nonsense mutations found in 10%–15% of DMD patients [29]. Ataluren (Translarna, PTC124) has been suggested to promote near‐cognate tRNA insertion at PTCs, allowing ribosomal read‐through and production of functional protein [30]. Although conditionally approved by EMA in 2014, it lost approval in 2024 due to limited efficacy and an unfavourable benefit–risk profile. Overall, PTC read‐through drugs have yet to gain approval, and data on their cardiac effects remain scarce.

### 2.2 DNA Modulation. Gene Therapies

While mRNA modulation has emerged as a clinically validated approach to restore dystrophin expression in DMD, its transient effects require repeated administration. In contrast, DNA‐targeting strategies hold promise for more durable correction of the underlying genetic defect by either editing the endogenous gene or introducing stable transgene expression. These approaches include genome editing techniques such as CRISPR‐Cas9, as well as gene replacement using shortened dystrophin transgenes (e.g., microdystrophin).

AAV‐based systems are a top choice for gene therapy due to their low immunogenicity, efficient transduction of non‐dividing cells and sustained transgene expression. Naturally occurring AAV Serotypes 6, 8 and 9 exhibit tropism for striated muscles, with AAV9 showing superior cardiac delivery via systemic administration [31, 32]_._ However, several challenges remain, including toxicity from off‐target effects and pre‐existing immunity.

To address these issues, newly engineered myotropic AAV variants such as AAVrh74 (closely related to AAV8) [33], as well as AAV9‐derived MyoAAV [34] and AAVMYO [35], have been developed to enhance muscle‐specific transduction. AAVrh74, derived from rhesus monkeys, may avoid pre‐existing immunity associated with human AAV serotypes like AAV9, as it has a low seroprevalence (15%–20%) in DMD patients and has shown a relatively low risk of adverse events in patients, making it a promising candidate for gene therapy [33]. AAVrh74 has been combined with the muscle‐specific MHCK7 regulatory region to drive expression of the SRP‐9001 microdystrophin transgene. The MHCK7 comprises an α‐myosin heavy chain (α‐MHC) enhancer and a muscle creatine kinase (MCK) enhancer/promoter, enabling robust expression in both skeletal and cardiac muscle while minimizing off‐target expression [33].

MyoAAV and AAVMYO, both engineered variants, share the arginine–glycine–aspartic acid (RGD) motif, which governs their muscle tropism by interacting with integrins on muscle cells, including cardiomyocytes, and may reduce liver uptake [36]. MyoAAV 1A and 2A provide greater potency and selectivity than AAV9 and AAVrh74, enabling lower dosages to achieve therapeutic efficacy [34]. In recent comparative in vivo characterization, MyoAAVs 2A and 4A showed higher targeting of hind limb and cardiac muscle compared with both AAV9 and AAVMYO, the latter being most efficient in the diaphragm [36].

Despite these advances, safety concerns become more pronounced when targeting up to 40% of total body mass in DMD, as high vector doses are necessary to effectively reach both skeletal muscles and the heart [37]. rAAV vectors (5 × 10^13^–2 × 10^14^ vg/kg) have led to events such as cardiopulmonary insufficiency and thrombocytopenia, including reported patient death [38].

#### 2.2.1 CRISPR‐Cas9 Gene‐Editing Approaches

Traditional DMD therapies, such as exon skipping, target specific mutations but have limited scope. In contrast, CRISPR‐Cas9 offers broader potential by enabling mutation‐specific editing across various dystrophin mutations. Unlike ASOs that require continuous administration, CRISPR‐Cas9 promises a single, durable treatment. Key strategies include exon excision, skipping, reframing, knock‐in, base editing and prime editing [37] (Figure 3). Notably, ‘myoediting’ uses optimal guide RNAs to target 12 key exons in *DMD*, correcting large deletions, point mutations or duplications through single‐site genome editing in hiPSCs [39].

Translational models have confirmed CRISPR‐Cas9 somatic genome editing improves DMD pathology in a DMDΔ52 pig model and patient‐derived hiPSC model [40]. In vivo administration of AAV9 carrying an intein‐split Cas9 and guide RNAs flanking Exon 51 (AAV9‐Cas9‐gE51) produced a truncated dystrophin variant (DMDΔ51–52) and improved skeletal muscle function after intramuscular injection. Systemic delivery resulted in widespread dystrophin expression in skeletal muscle, diaphragm and heart of DMD pigs, extending survival and reducing arrhythmogenic risk. Similarly, in cardiomyocytes from DMDΔ52 hiPSCs, AAV6‐Cas9‐g51 restored dystrophin expression and ameliorated abnormal calcium handling and arrhythmogenic susceptibility [40].

As a derivative of CRISPR/Cas9, base editing enables precise nucleotide changes without inducing double‐strand breaks. Adenine base editing (ABE), for instance, converts adenine to guanine. Systemic ABE delivery in humanized DMD mice restored cardiac dystrophin expression to 78.38% of levels observed in wild‐type mice [41]. More complex edits, such as insertions, deletions and precise nucleotide corrections, are enabled by prime editing. This method uses a prime editing guide RNA (pegRNA) and reverse transcriptase to introduce targeted changes directly into the DNA. For example, in hiPSC‐CMs, prime editing corrected the ΔEx51 mutation by reframing Exon 52, restoring dystrophin up to 39.7% and normalizing calcium cycling and arrhythmias [42].

Despite advances, challenges remain before clinical use of CRISPR‐based systems. Delivery efficiency and the needed high AAV doses pose hurdles. AAV packaging limitations often necessitate dual‐AAV systems for in vivo delivery of CRISPR components. Safety concerns emerged in 2023 following the death of a 27‐year‐old DMD patient, who received high‐dose (1 × 10^14^ vg/kg) rAAV9–dSaCas9 (‘dead’ *Staphylococcus aureus* Cas9, with inactivated nuclease activity) [38]. The patient developed severe cardiopulmonary toxicity, including pericardial effusion and acute respiratory distress syndrome (ARDS), attributed to cytokine‐mediated capillary leak syndrome, likely exacerbated by high pulmonary vector accumulation and limited physiological reserve associated with advanced disease [38].

Translating CRISPR therapies requires addressing off‐target effects and immune responses to AAV vectors and Cas proteins [37]. Pre‐existing immunity to AAV capsids and Cas9 can limit patients' eligibility or therapeutic efficacy [37, 43], while readministration is hindered by neutralizing antibodies against AAV, necessitating alternative serotypes or immunosuppression (e.g., corticosteroids, plasmapheresis) [37]. Strategies to reduce Cas9 immunogenicity include the development of hypoimmunogenic variants, regulatory T cell–based tolerance strategies and tissue‐specific or transient expression systems [43]. Managing these immune challenges is crucial for safe, durable genome editing in DMD.

#### 2.2.2 Dystrophin‐Based Gene Therapy

Gene therapy for DMD faces challenges due to the large size of the dystrophin gene, mutation variability and the need to target skeletal and cardiac muscles. To overcome the limitations posed by the size of *DMD* cDNA, a functional analysis of dystrophin domains was performed, revealing that selective deletions in different regions of the protein can yield functional minidystrophins and microdystrophins, which have shown promising results in rodent and canine models [44, 45]. Preclinical studies showed that rAAVrh74.MHCK7.microdystrophin administration resulted in significant functional improvements in *mdx* mice [46] and dystrophic rats [47]. In dystrophic rats, this therapy restored mobility, significantly improved cardiac function and extended median survival beyond 25 months, with microdystrophin detectable in both skeletal muscle and the heart at 2, 24 and 52 weeks post administration [47].

**FIGURE 4:**
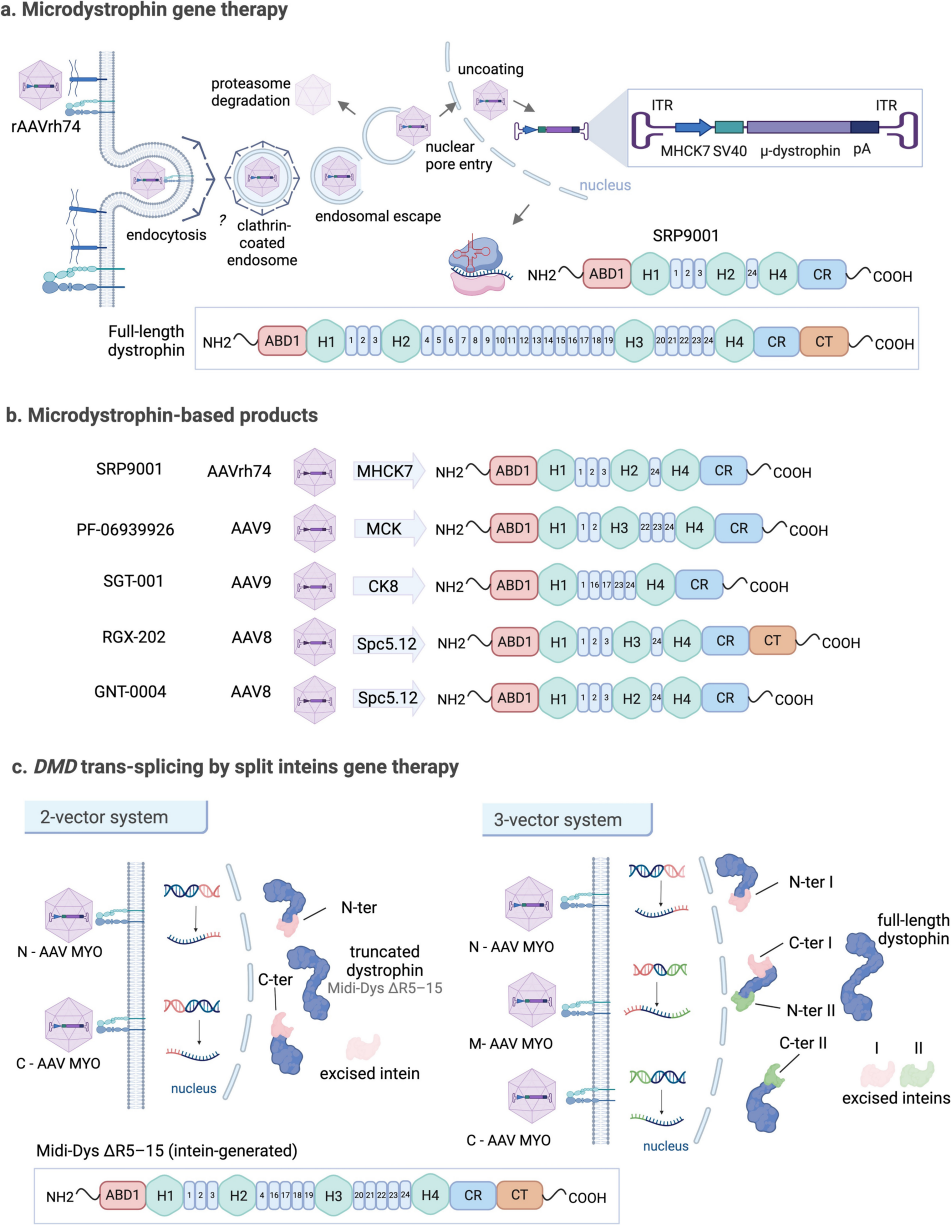
Dystrophin‐based gene therapy strategies. (a) Elevidys (SRP‐9001) uses a recombinant AAVrh74 vector to deliver a microdystrophin transgene into muscle cells. The expressed microdystrophin lacks CT domain, H3 region and SR 4–23 from the full‐length dystrophin. (b) Microdystrophin therapies in clinical trials retain key structural domains like ABD1, CR and a shortened rod domain, ensuring functional integrity. These microdystrophins are delivered using viral vectors optimized for efficient gene transfer and tissue‐specific expression. (c) The AAVMYO system overcomes AAV size limitations with intein‐mediated trans‐splicing, using a two‐vector (N‐ and C‐AAVMYO) or three‐vector system (N‐, M‐ and C‐AAVMYO) to produce truncated or full‐length dystrophin.

Based on clinical studies of this gene therapy, the FDA granted accelerated approval in June 2023 to delandistrogene moxeparvovec (Elevidys; Sarepta Therapeutics), the first gene therapy for DMD. Elevidys delivers SRP‐9001 microdystrophin via a single‐dose AAVrh74 vector [45, 48] (Figure 4a and Tables 1 and S1). Approval was based on Phase 2 trial data (NCT03769116) demonstrating increased microdystrophin expression in the gastrocnemius muscle, a surrogate endpoint [49]. Despite the lack of supporting Phase 3 data and considerable deviations from standard clinical and laboratory practices [9], the FDA expanded Elevidys' approval in June 2024 to include both ambulatory and nonambulatory patients. Although the Phase 3 trial (NCT05096221) did not meet its primary endpoint (North Star Ambulatory Assessment, NSAA), secondary and exploratory endpoints, such as timed function tests and creatine kinase levels, suggested clinical benefit [50]. Due to safety concerns, Sarepta paused Elevidys shipments for nonambulatory patients in June 2025 after reports of two deaths among nonambulatory recipients and a third in a trial for a different dystrophy using the same AAVrh74 vector. On 21–22 July 2025, at the FDA's request, all shipments were paused. Following FDA recommendations, on 28 July 2025, the voluntary hold for ambulatory patients was lifted and shipments resumed.

In addition to Elevidys, other microdystrophin constructs are being evaluated in clinical trials (Figure 4b). Among them, Pfizer's investigational therapy, fordadistrogene movaparvovec (PF‐06939926) has been paused following serious adverse events, including dehydration, kidney injury and a fatal event reported in the Phase 2 DAYLIGHT trial (NCT05429372), which assessed safety and tolerability in 10 boys with DMD aged 2–3 years. Consequently, Pfizer has also paused dosing in the crossover phase of the Phase 3 CIFFREO trial (NCT04281485) involving boys aged 3–7 years.

The emergence of molecular and gene‐targeted therapies for DMD, including ASOs (e.g., eteplirsen) and gene therapy (delandistrogene moxeparvovec), has gained accelerated or conditional approval despite limited clinical data [51]. While increasing access, concerns about long‐term efficacy, postmarketing surveillance and high costs underscore the need for longitudinal evidence and equitable reimbursement policies [51].

To overcome the challenge of the large size of the *DMD* gene, protein trans‐splicing (PTS) via split inteins offers a promising alternative strategy for restoring dystrophin expression [52] (Figure 4c). The transcript can be divided into smaller segments, each packaged in myotropic AAVs (e.g., AAVMYO or MyoAAV4A), and reassembled via PTS into either a truncated (midi‐Dys; two‐vector system [52]) or a full‐length protein via a three‐vector system [52, 53] (Figure 4c). In *mdx*4cv mice, a triple‐vector approach restored full‐length dystrophin expression in both skeletal and cardiac muscle, demonstrating functional advantages over microdystrophins currently in clinical trials [52, 53]. Notably, efficacy was achieved at relatively low vector doses (2 × 10^13^ vg/kg), with safety still to be assessed.

Besides dystrophin‐targeted therapies, strategies addressing the pathophysiological consequences of dystrophin deficiency by targeting muscle‐stabilizing proteins, such as follistatin, beta‐1,4‐galactosyltransferase 2 (GALGT2), biglycan [20] and cardiac dysfunction, are being explored. For example, AAV9‐mediated sarco/endoplasmic reticulum calcium ATPase 2 (SERCA2a) gene therapy reduced calcium overload and improved muscle and heart function in *mdx* mice [54] but requires validation in larger mammals.

Fibrosis, a major DMD progression factor, affects skeletal muscle early, while cardiac fibrosis appears before age 10 and progressively worsens as the disease advances [55]. In 2024, FDA‐approved Duvyzat (givinostat), a histone deacetylase (HDAC) inhibitor that has shown potential in reducing inflammation and fibrosis while supporting muscle repair [56].

Pharmacological interventions also address systemic DMD aspects. Idebenone, a mitochondrial antioxidant, slowed respiratory decline in steroid‐naïve patients, especially younger ones, in the DELOS trial [57]. Vamorolone, a dissociative corticosteroid, improved motor function while minimizing glucocorticoid‐associated side effects [58], showing a milder impact on growth and bone health, supporting its potential as a safer long‐term option in DMD [58].

Collectively, these gene‐targeted and disease‐modifying strategies hold transformative potential for DMD treatment, yet their long‐term safety, efficacy and accessibility remain critical challenges to be addressed in future clinical research.

## 3 hiPSCs for DMD Cardiac Modelling and Preclinical Testing

Although animal models have advanced DMD research, they fall short of accurately replicating human disease. The commonly used *mdx* mouse carries a nonsense mutation in Exon 23 and shows mild symptoms with late‐onset cardiomyopathy, unlike the progressive human DMD [59]. More severe phenotypes, such as in D2‐mdx mice or various chemical and genetic mdx variants, have been developed to better model disease progression [5]. Some models, like the mdx: Utrn^−^/^−^ double knockouts, combine additional mutations to further accelerate disease pathology [5]. In contrast, large animal models such as dystrophic pigs and golden retrievers resemble human DMD more closely but are expensive and show variability due to genetic background, small cohort sizes and mosaicism in engineered lines. Non‐human primates, including CRISPR‐edited macaques, exhibit early muscle degeneration and cardiac defects but do not fully mimic DMD progression [59]. These limitations, including species differences and incomplete cardiac modelling, highlight the need for human‐specific platforms.

**FIGURE 5:**
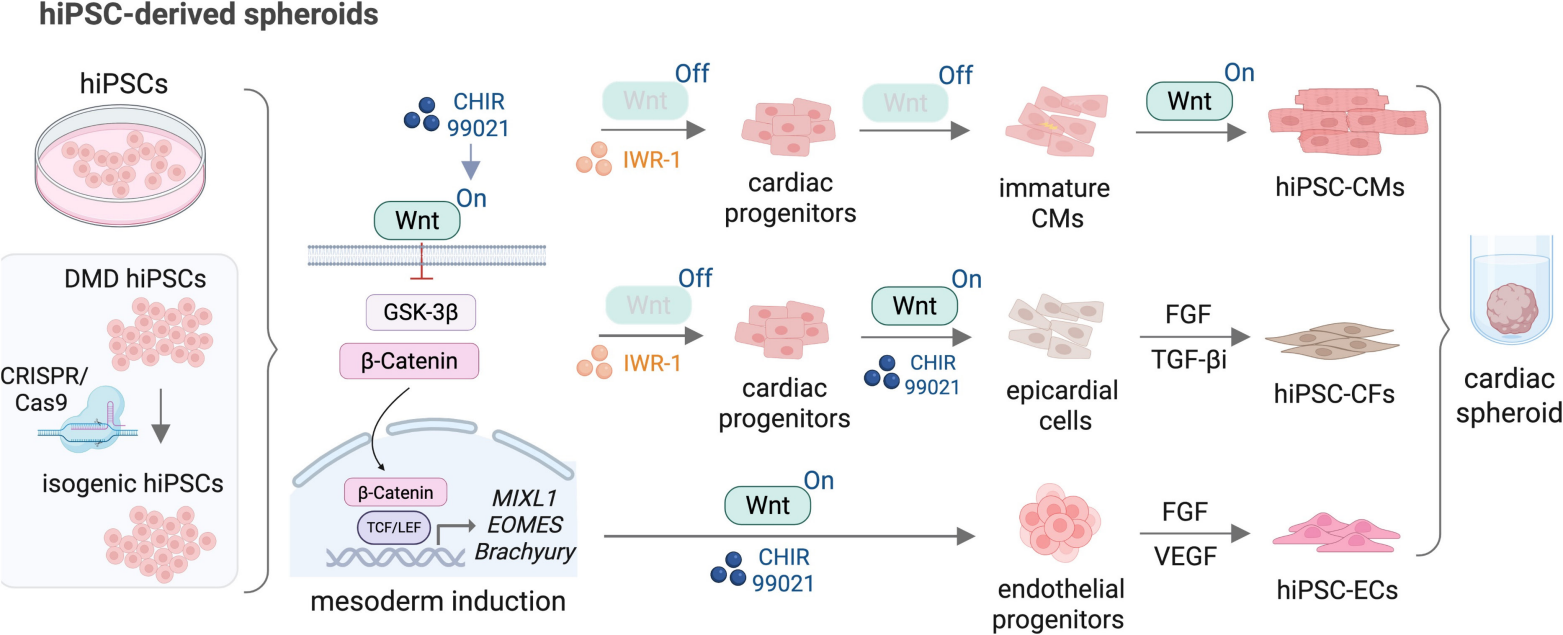
hiPSC differentiation and generation of hiPSC‐derived spheroids. Differentiation of hiPSCs into cardiomyocytes (hiPSC‐CMs), cardiac fibroblasts (hiPSC‐CFs) and endothelial cells (hiPSC‐ECs) begins with mesoderm induction through Wnt signalling activation by CHIR99021. Inhibition of Wnt signalling via IWR‐1 forms cardiac progenitors, which can either form immature and then mature cardiomyocytes or, if treated again with CHIR99021, form epicardial cells, which differentiate to cardiac fibroblasts in the presence of FGF and transforming growth factor beta 1 inhibitor. hiPSC‐ECs are generated by use of CHIR99021 to induce endothelial progenitors, which are then treated with FGF and VEGF for endothelial cell differentiation.

Since 2007, hiPSCs [60] have enabled patient‐specific disease modelling, preserving unique genetic backgrounds and overcoming both interspecies differences and ethical concerns associated with human embryonic stem cells (hESCs). CRISPR–Cas9‐edited isogenic pairs of diseased and corrected control cells [61] provide a consistent genetic background for studying DMD pathogenesis in human cells (Figure 5). hiPSC‐CMs from DMD patients exhibit dystrophin loss, abnormal calcium handling, mitochondrial damage, increased ROS, dysregulated iron metabolism and altered electrophysiology [62, 63, 64], supporting their use in preclinical therapy testing. Recent quantitative approaches enable high‐throughput, longitudinal tracking of single‐cell contractile dynamics in hiPSC‐CMs [65].

Beyond modelling typical DMD phenotypes, hiPSCs from female carriers serve as an important human‐based model to investigate the effects of dystrophin mosaicism on cardiac pathophysiology [15]. Functionally, female DMD hiPSC‐CMs display arrhythmias, delayed afterdepolarizations, prolonged action potential duration (APD) and increased beat rate variability, which are not observed to the same extent in male DMD hiPSC‐CMs [15]. These observations support the use of female carrier‐derived hiPSC‐CMs as a distinct model of cardiac involvement in DMD.

While hiPSC‐CMs offer valuable insights into DMD cardiac pathology, their immature, embryonic‐like phenotype limits direct clinical translation. Despite prolonged culture, hormonal cues, endothelial coculture, optimized matrices and biophysical stimulation, fully adult‐like characteristics remain difficult to achieve [66]. Advanced 3D hiPSC‐derived cardiac organoids (COs) not only promote maturation in vitro and after implantation in immunodeficient mice but also recapitulate myocardial architecture and microenvironments better, providing superior platforms for studying cell interactions and disease mechanisms compared with traditional 2D cultures [67]. These emerging 3D models represent a promising direction for more physiologically relevant DMD cardiac research.

## 4 3D Cardiac Models

**FIGURE 6:**
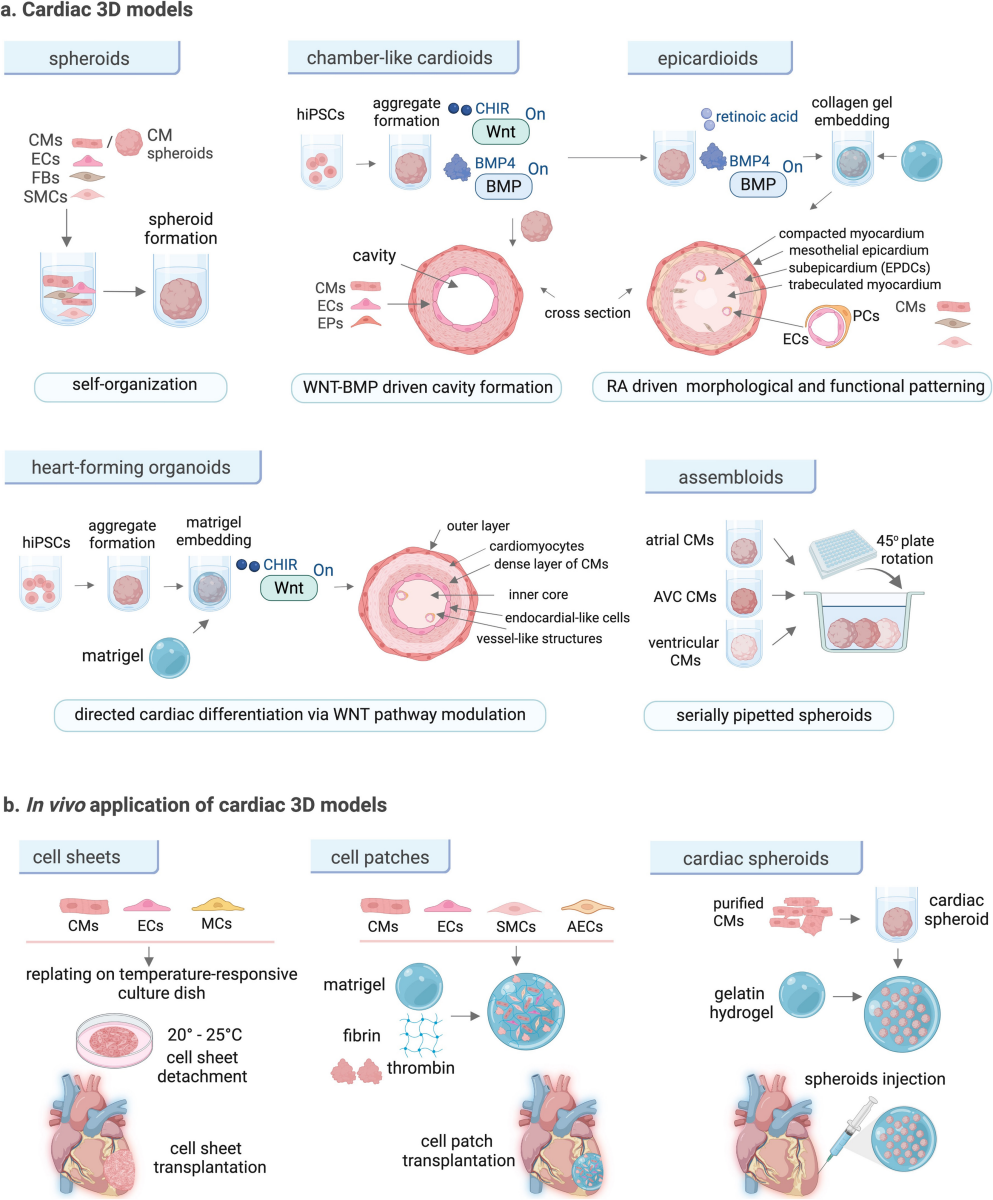
Cardiac 3D models and in vivo applications. (a) Simplified generation and structure of hiPSC‐derived cardiac 3D models include self‐organizing spheroids composed of cardiac‐specific cells in various ratios to mimic cellular interactions. Cardioids, epicardioids and heart‐forming organoids (HFOs) form through hiPSC aggregation and directed differentiation with Wnt pathway modulation. HFOs and cardioids reveal self‐organizing features of cardiogenesis, providing insights into heart development. Epicardioids replicate epicardial and myocardial compartments with retinoic acid (RA)–dependent self‐patterning. Assembloids combine multiple spheroids to create complex cardiac structures, for example, mimicking the heart's conduction axis. (b) In vivo, cell sheets made of CMs, ECs and mural cells are transplanted onto the heart's surface for scaffold‐free integration. Cell patches, composed of CMs, ECs, SMCs and arterial endothelial cells (AECs), are embedded in a matrigel, fibrin and thrombin matrix and applied to damaged cardiac tissue to support regeneration. Cardiac spheroids, formed from purified CMs, are suspended in gelatin hydrogel and delivered via a device for precise placement in injured heart regions to restore function.

Building upon hiPSC‐CMs, 3D cardiac models, typically self‐organizing or formed within moulds, offer enhanced structural and functional fidelity, enabling detailed investigation of DMD‐associated cardiac pathology in a more physiologically relevant context. Beyond cardiomyocytes, recent models include hiPSC‐derived cardiac fibroblasts (hiPSC‐CFs) [68, 69, 70], mouse embryonic fibroblasts [71], human dermal fibroblasts [72], human fetal cardiac fibroblasts [73], hiPSC‐derived endothelial cells (hiPSC‐ECs) [68, 69, 70, 74] (Figure 5), human umbilical vein endothelial cells, skeletal muscle cells [75] or epicardial cells [74], to enhance complexity and function (Figure 6a). With variations in composition and cell type ratios, these models are referred to by diverse names, including COs [61, 76, 77, 78], vascularized COs (VCOs) [74], cardiac spheroids [73, 75, 79, 80], cardioids [81], epicardioids [82] and cardiac microtissues (cMTs). The simplest method to create human heart spheroids involves self‐assembly of hiPSCs into embryoid bodies (EBs) via modulation of Wnt signalling, modelling fetal heart development and generating all major cardiac lineages [78]. These spheroids beat robustly within a week and maintain contractile activity for up to 8 weeks [78].

3D structures show more mature metabolism than 2D cultures, with enhanced fatty acid oxidation, glucose utilization and mitochondrial function [83]. The importance of cellular complexity for cardiac model maturation has been emphasized [68, 77, 84]. In a 3D culture system, the inclusion of hPSC‐ECs and prolonged culture enhanced hPSC‐CMs maturity, as evidenced by the upregulation of genes related to sarcomere assembly, ion channel function and calcium handling, along with improved electrophysiological properties and more mature action potentials [85]. Coculturing hiPSC‐CMs with fetal cardiac fibroblasts enhanced tissue‐like features and beat rate [73].

Tricellular cMTs containing hiPSC‐CMs, hiPSC‐CFs and hiPSC‐ECs, were established by Giacomelli et al. [69]. hiPSC‐CMs in cMTs with hiPSC‐CFs showed better sarcomeric structures, T‐tubules and enhanced contractility compared with cMTs without hiPSC‐CFs. Electrophysiological maturation was driven by hiPSC‐CMs and hiPSC‐CFs interactions through Connexin 43 (CX43) gap junctions and increased cyclic adenosine monophosphate (cAMP) [69]. These tricellular models support hiPSC‐CM maturation and enable study of cardiovascular complications [70] with application of isogenic disease and control cell sets [68]. Four‐cell (C4) spheroids including hiPSC‐derived smooth muscle cells (SMCs), spontaneously produced ECM, maintained beating activity for 60 days and showed improved CM energetics and reduced apoptosis [75]. These results support the hypothesis that hiPSC‐CMs maturation is enhanced in a 3D spheroid culture, where hiPSC‐ECs, hiPSC‐CFs and hiPSC‐SMCs promote electrical activity [75].

To generate a cardiac model with an epicardial compartment, epicardioids were created using retinoic acid (RA), a key factor involved in cardiac anteroposterior patterning and epicardial development [82] (Figure 6a). RA has been shown to promote the differentiation of hPSCs into epicardial cells in vitro [86]. hiPSC‐derived epicardioids exhibited RA‐dependent self‐organization of both myocardium and epicardium. Lineage tracing and multiomic profiling uncovered the potential of mesothelial epicardial cells in epicardioids to differentiate into different cardiac lineages [82]. While epicardioids replicate the epicardial and myocardial compartments, other models like heart‐forming organoids [87] or cardioids reveal self‐organizing features and replicate early heart development [81, 87, 88]. Additionally, cardiac assembloids fuse different spheroids to mimic atrioventricular conduction axis simulating conduction patterns observed in vivo [89] (Figure 6a).

COs offer a relevant 3D environment to study multicellular interactions and disease progression but pose challenges for functional assessment, including force measurement, imaging and electrophysiological analysis. While 3D imaging allows visualization of tissue architecture, cellular composition and interactions [90], conventional 2D microelectrode arrays (MEAs), typically used to assess electrical activity, are limited to a single plane and fail to capture signals across the entire organoid [91]. To address this, a programmable, shape‐adaptive shell MEA has been developed, enabling full‐surface electrical mapping, conduction velocity analysis and calcium imaging to validate electrophysiological patterns [92].

To advance cardiac tissue engineering and disease modelling, biomimetic strategies that incorporate scaffolds are being developed to replicate the heart's mechanical, electrical and biochemical properties, including ECM‐mimicking architectures and features that promote cell–cell interactions [93]. In cardiac regeneration, fibrin‐based engineered heart tissues (EHTs) [94], decellularized heart matrices [95, 96] and cardiac cell patches [97] support tissue repair and enhance cardiac function.

### 4.1 Comparison of 3D Cardiac Models—Suitability for Drug Screening and Preclinical Testing

Self‐organizing models, such as spheroids, cMTs and COs, offer unique disease and therapeutic insights not achievable in 2D systems (Table 2). Cardiac spheroids [73, 75, 79, 80] and cMTs [68, 69, 70, 83, 85, 98] provide robust contractile readouts, in which the inclusion of CFs enhances electrophysiological maturity and enables modelling of hypertrophy or fibrosis. COs [61, 76, 77] may support cardiotoxicity testing and drug discovery via direct CM functional readouts (contractility and calcium imaging) with fewer non‐CM variables. Cardioids [81] model early cardiac morphogenesis and form chamber‐like cavities, making them preferable for developmental disease studies and high‐throughput pharmacological screening. Epicardioids [82] reveal epicardium–myocardium crosstalk, EMT and pathological remodelling (hypertrophy/fibrosis) but require complex protocols and retain a fetal‐like epicardial phenotype.

**TABLE 2:** Comparison of 3D cardiac models

3D model nomenclature	Used cell types or their combinations	Key features	Proposed application	Strengths	Limitations	Key references
Cardiac organoids (COs)	hESC‐CMs hiPSC‐CMs	▪ Self‐organizing ▪ Early heart phenotype; immature cardiomyocytes predominate ▪ Long‐term beating (even up to 90 days)	♦ Cardiotoxicity studies ♦ Drug discovery ♦ Disease modelling ♦ Hypertrophy/fibrosis studies	✓ Analysis of CM‐restricted function ✓ Reduced variability from non‐CM cell types (fewer cofounders) ✓ Direct functional readouts from CMs (contractility, Ca^2+^) ✓ Reproducibility and scalability across cell lines	✗ Resemble fetal heart ✗ Lack vascularization ✗ Variability between batches	[61, 74, 76, 77, 78]
Cardiac spheroids/cardiac microtissues (cMTs)	hiPSC‐CMs hiPSC‐CFs hiPSC‐ECs hiPSC‐SMCs Human embryonic CFs	▪ Self‐organizing ▪ Based mostly on hiPSC‐derived cells ▪ Spontaneous beating (from 3–4 days after seeding) with physiological responses ▪ Electrophysiologically mature features enhanced by CFs	♦ Functional studies ♦ Hypertrophy/fibrosis studies ♦ Disease modelling ♦ Preclinical cardiac therapy studies ♦ Drug screening	✓ Functional readouts (contractility) ✓ Electrophysiological maturity ✓ Demonstrated in vivo potential (cardiac spheroids) ✓ Improve CMs maturation (sarcomere structure, contractility and mitochondrial function) via cell–cell interactions ✓ Reproducibility and scalability across cell lines	✗ Resemble fetal heart ✗ Moderate tissue complexity ✗ Lack vascularization ✗ Variability between batches	[68, 69, 70, 73, 75, 79, 80, 83, 85, 98]
Cardioids	hPSC‐CMs hPSC‐SMCs hPSC‐CFs	▪ Morphogen‐guided self‐organization ▪ mimic early cardiac morphogenesis ▪ chamber‐like cavities	♦ Early heart development studies ♦ Modelling multichamber formation ♦ Genetic and morphogenesis studies	✓ Embryonic patterning ✓ Chamber‐like structures ✓ Functional studies (contraction and electrophysiology) ✓ Reproducibility and scalability across cell lines	✗ Resemble early fetal heart ✗ Lack vascularization ✗ Variability between batches ✗ Early‐stage focus	[81]
Epicardioids	hPSC‐derived epicardial cells hPSC‐vSMCs hPSC‐CFs	▪ Retinoic acid (RA)‐driven patterning—form epicardial and myocardial layers ▪ Mesothelial potential	♦ Dissect epicardium–myocardium crosstalk during human ventricular development ♦ Hypertrophy/fibrosis studies	✓ Include a functional epicardial layer ✓ Epicardium‐driven myocardial compaction/maturation yields advanced tissue self‐organization ✓ Capture EMT	✗ Complex protocols ✗ Lack vascularization ✗ Variability between batches ✗ Resemble fetal epicardium	[82]
Cardiac assembloids	Atrial CM atrioventricular canal CMs Ventricular CMs	▪ Technically demanding assembly of three types of spheroids ▪ Recapitulates atrioventricular conduction patterns	♦ Complex conduction disorders studies (molecular, cellular and functional causes)	✓ Model atrioventricular block ✓ Reproduce conduction delay (fast–slow–fast) ✓ Unidirectional impulse propagation	✗ Narrow application scope ✗ Demanding assembly ✗ Heterogeneity in the composition of AV nodal cardiomyocytes, which include transitional cells extending both in the direction towards the atria and towards the ventricles	[89]
Engineered heart tissues (EHTs)	hESC‐CMs hESC‐AECs hiPSC‐CMs hiPSC‐CFs hiPSC‐ECs hiPSC‐SMCs	▪ Scaffold‐supported contractile tissues ▪ Mimic force generation and native heart microenvironment	♦ Contractility studies ♦ Drug screening ♦ Tissue engineering	✓ Enable mechanical force measurements ✓ Customizable geometry ✓ improved functional and structural phenotype	✗ Complex and costly fabrication ✗ Lower physiological relevance in self‐organization ✗ Potential issues with electrical coupling and arrhythmia risk	[94]

Abbreviations: CFs—cardiac fibroblasts, CMs—cardiomyocytes, cMTs—cardiac microtissues, COs—cardiac organoids, ECs—endothelial cells, EHTs—engineered heart tissues, EMT—epithelial‐to‐mesenchymal transition, hESC‐AECs—human embryonic stem cell–derived arterial endothelial cells, hESC‐CMs—human embryonic stem cell–derived cardiomyocytes, hiPSC‐CFs—human‐induced pluripotent stem cell–derived cardiac fibroblasts, hiPSC‐CMs—human‐induced pluripotent stem cell–derived cardiomyocytes, hiPSC‐ECs—human‐induced pluripotent stem cell–derived endothelial cells, hiPSCs—human‐induced pluripotent stem cells, hiPSC‐SMCs—human‐induced pluripotent stem cell–derived smooth muscle cells, hPSC‐CFs—human pluripotent stem cell–derived cardiac fibroblasts, hPSC‐CMs—human pluripotent stem cell–derived cardiomyocytes, hPSC‐SMCs—human pluripotent stem cell–derived smooth muscle cells, hPSC‐vSMCs—human pluripotent stem cell–derived vascular smooth muscle cells, RA—retinoic acid, (v)SMCs—(vascular) smooth muscle cells.

All models resemble the fetal heart, offer moderate tissue complexity, lacking vascularization and innervation. While some include ECs, functional, perfusable vasculature is absent, limiting maturation and scalability. In addition, batch‐to‐batch variability needs to be considered due to differences in cell quality and culture conditions. Among current options, assembloids show the highest spatial organization, modelling interregional electrical signalling. By combining distinct cardiac domains, they enable a precise investigation of complex electrophysiological processes. Cardiac assembloids [89] recapitulate atrioventricular signal transmission and allow mechanistic analysis of complex conduction disorders. However, their utility remains limited by a narrow scope and technically challenging fabrication. EHTs [94], scaffold‐supported contractile constructs, enable calibrated force measurements and customizable geometry for contractility studies, drug screening and tissue engineering. Despite improved structure and function, their application is limited by complex set‐up, reduced self‐organization and occasional electrical‐coupling/arrhythmia issues. Key features and limitations of 3D cardiac models are summarized in Table 2.

### 4.2 In Vivo Use of PSC‐Derived Models for Cardiomyocyte Engraftment

The occurrence of engraftment arrhythmias, associated with the immature electrophysiological profile of hiPSC‐CMs, has been reported already in large animal models, including pigs [66] and macaques [99]. Advanced approaches such as cell sheets [100], patches [101, 102] (Figure 6b) and 3D cardiac constructs address limitations of dissociated cardiomyocytes, including poor retention and arrhythmogenic risk after transplantation [66].

hiPSC‐engineered cardiovascular tissue sheets (hiPSC‐CTSs) enhanced cardiac function in infarcted rats without causing fatal arrhythmias [100]. Similarly, a clinical trial using hiPSC‐CMs patches in ischemic cardiomyopathy showed symptom improvement without adverse events [101]. Patch safety and efficacy were also validated in a porcine model [102]. Nevertheless, challenges such as suboptimal electrical integration and arrhythmogenic potential remain in certain contexts. In 3D model, incorporation of supporting cell types improved tissue organization and electromechanical coupling [103]. Elevated expression of junctional proteins such as CX43 in hiPSC‐CMs and hiPSC‐CFs reduced ectopy and re‐entry circuits, key mechanisms underlying arrhythmias [104].

A recent strategy combined hiPSC‐derived cardiac spheroids with gelatin hydrogel and a transplant device (Figure 6b), optimizing distribution and retention of hiPSC‐CMs during epicardial injection, improving function in a porcine heart failure model [105]. Although xenograft‐associated inflammation limited retention, heart function improved at 8 weeks with no teratomas observed [80]. However, ventricular arrhythmias occurred post transplant, correlating with immune rejection, but subsided as graft size decreased [80]. Pharmacological treatments (amiodarone and ivabradine) partially reduced arrhythmic events. Combining these treatments with less arrhythmogenic cell populations may offer a strategy to enhance posttransplant safety [106].

In addition to arrhythmogenic risks, posttransplant challenges such as immune rejection and limited graft survival remain major barriers. In preclinical models, stable engraftment of hiPSC‐CMs requires immunosuppressive regimens to limit immune rejection and promote graft survival [107]. In a porcine infarct model, 1 × 10^9^ hESC‐CMs yielded grafts occupying approximately 15% of the scar, underscoring the need for dose scaling and enhanced graft retention and survival in human‐sized hearts. Improved outcomes reflected the combination of rigorous, closely monitored multiagent immunosuppression (calcineurin inhibitor, T‐cell costimulatory blockade and corticosteroid) and cell prosurvival interventions (precryopreservation heat‐shock and delivery in a prosurvival cocktail), thereby enhancing posttransplant survival and graft retention [107].

### 4.3 3D Cardiac Models in DMD Research

Building on advances in 3D cardiac modelling, new strategies are now applied to better capture disease complexity and promote CM maturation in DMD models, offering more physiologically relevant systems for studying disease progression and evaluating therapeutic interventions.

Duelen et al. developed EHT constructs from DMD patient–derived hiPSC‐CMs, demonstrating maturation of cardiomyocytes in a 3D system, including enhanced expression of cardiac‐specific genes such as *MYL2* (myosin light chain II) and *TNNI3* (cardiac troponin I). These DMD‐EHTs recapitulated disease phenotype, exhibiting impaired contractility and increased reactive oxygen species (ROS) production, associated with elevated NADPH oxidase 4 (NOX4) activity [108]. By mixing DMD hiPSC‐CMs with genetically corrected counterparts in varying proportions in EHTs, it was demonstrated that inclusion of 30% corrected cardiomyocytes partially improved contractile function, while 50% resulted in maximal phenotypic rescue [39]. This aligns with prior studies on *mdx* mosaic mice [11, 12], suggesting that a 50% cardiac dystrophin correction may be a sufficient therapeutic target. However, data from female DMD carriers, who exhibit natural dystrophin mosaicism, indicate significant cardiac dysfunction despite ~50% dystrophin‐positive cells, highlighting the need for therapies that target broader correction to ensure sustained cardiac stability.

EHTs have also been developed from CRISPR‐edited hiPSC‐CMs expressing truncated dystrophin lacking part of the actin‐binding domain [109]. This DMD EHT model effectively replicated disease phenotype aspects such as decreased contractility, shorter sarcomeres, changed Ca^2+^ transients, increased beat rate irregularity and transcriptome disturbances. Compared with isogenic controls, genes associated with calcium homeostasis and ECM organization were downregulated, while genes linked to membrane potential regulation, cardiac muscle growth and contraction were upregulated. The EHT system provided sufficient mechanical loading and maturation cues to unmask disease‐relevant phenotypes that were absent in 2D cultures. Importantly, these deficits, including impaired contractility, abnormal Ca^2+^ handling, shorter sarcomeres and beat rate irregularities, were detected within 3 weeks, whereas similar features in *mdx* mice appear only at older ages [109].

To further explore disease progression in long‐term 3D cultures, Marini et al. cultured DMD patient–derived COs for up to 93 days [61]. These COs showed progressive deterioration of cardiac function, including hypertrophy, dilation, fibrosis and adipose tissue accumulation. Over time, these structural changes closely mirrored the pathological remodelling in DMD hearts [61]. Furthermore, DMD COs showed impaired ryanodine receptor 2 (RyR2)–driven Ca^2+^ signalling compared with isogenic and healthy controls, indicating disrupted calcium homeostasis. From Day 14, a gradual loss of α‐, β‐, γ‐ and δ‐sarcoglycans emerged. This loss of DAGC components correlates with increased mechanical stress on cardiomyocytes and potentially accelerates fibrosis. Additionally, increased NOX4 expression in DMD COs highlighted the role of oxidative stress in disease progression [61].

A recent study [110] demonstrated the use of patient‐derived COs as a rapid and scalable preclinical platform for evaluating both existing and novel ASOs in DMD. In COs from a patient lacking Exons 46–53, two Exon 45‐skipping ASOs, one FDA‐approved and one in Phase 2 trials, restored dystrophin expression, improved Ca^2+^ handling and rescued contractility. Approximately 40% of CO cells incorporated the ASO, and Exon 45 skipping was detected in ~15% of DMD transcripts. The platform was also applied to two patients with deep intronic mutations creating a cryptic splice acceptor site, leading to pseudoexon inclusion and premature transcript termination. For these patients, two personalized ASOs targeting distinct sequences overlapping the pathogenic variant corrected splicing defects, restored dystrophin expression to ≥ 20% of healthy levels, normalized Ca^2+^ transients and recovered contractile function [110].

3D cardiac modelling has advanced, yet replicating the complex extracellular environment of dystrophic myocardium remains challenging. To address this, DystroGel, a hydrogel derived from decellularized cardiac ECM (dECM) of a porcine DMD model (1 day old and 4 months old), has been developed, preserving key ECM components such as collagen I and IV while eliminating cellular material [111]. Mechanical tests indicated fibrosis‐like stiffening in 4‐month‐old DMD hydrogels, while proteomics revealed dysregulation of contractility, ECM organization and neurotrophic signalling pathways. The hydrogel supported survival and function of fibroblasts, iPSC‐CMs and iPSC‐derived sympathetic neurons. When cultured in this matrix, iPSC‐CMs exhibited gene expression shifts towards fibrotic and immature phenotypes, and sympathetic neurons showed reduced neurite sprouting, collectively recapitulating structural and functional hallmarks of dystrophic heart tissue [111].

So far, none of the 3D DMD models, including EB‐based COs [61, 110], EHTs [39, 108, 109] or the DystroGel hydrogel platform [111], have incorporated hiPSC‐ECs. Although ECs have been introduced into non‐DMD cardiac models, they have not yet enabled the formation of fully functional vasculature. Still, their integration into DMD‐specific systems may support structural organization and disease modelling fidelity.

## 5 Premature State of DMD Cell Therapy

Despite advances in hiPSC‐based disease modelling, the clinical translation of cell therapies for DMD remains in its infancy, with substantial biological and technical barriers yet to be addressed.

In DMD, progressive cardiomyocyte loss leads to replacement by fibrotic, noncontractile tissue. The adult human heart has a limited capacity for regeneration, with cardiomyocyte turnover declining from approximately 1% per year at age 25 to just 0.45% by age 75 [1]. Under homeostatic conditions, renewal relies primarily on existing cardiomyocytes, showing minimal regenerative response following injury [2]. In DMD, dystrophin loss leads to increased cardiomyocyte damage, which overwhelms the heart's limited regenerative capacity and accelerates dysfunction.

Cell therapies for DMD aim to deliver dystrophin‐expressing cells, either donor‐derived or genetically corrected patient‐specific cells, capable of crossing vascular barriers, integrating into host myocardium and evading immune rejection. Although various cell types, including satellite cells, other muscle‐derived stem cells or myogenic progenitors, have been explored [20], the associated challenges and complexities indicate that cell‐based approaches in DMD are still far from being translated into effective clinical treatments (Figure S1).

Currently, intramuscular delivery is the most common approach but is limited by poor engraftment, low dystrophin expression, restricted cell migration and limited functional improvement. Even with repeated dosing, it fails to target approximately 40% of total body mass, producing only localized effects, and to reach key muscle groups like the diaphragm and heart. On the other hand, systemic administration struggles with endothelial transmigration. This ability has been ascribed to mesoangioblasts, vessel‐associated stem cells, able to differentiate into a variety of mesodermal cell types including muscle cells [112], as an advantage over satellite cells and myoblasts, which require direct injection. In mdx mice, mesoangioblasts modified with a human artificial chromosome (HAC) vector carrying the entire human dystrophin locus ameliorated the dystrophic phenotype [112]. However, a first‐in‐human intra‐arterial transplantation of mesoangioblasts in five DMD patients showed safety but minimal efficacy, with dystrophin restoration in only one case [113].

Future strategies may rely on iPSC‐derived myogenic progenitors or iPSC‐CMs expressing dystrophin from autologous corrected or allogeneic sources [114]. The promise of easily accessible autologous cells holds considerable potential and may inform future experimental designs. However, unresolved questions including immune responses to dystrophin [115] or the immunogenicity of allogeneic cells remain. Additionally, engraftment arrhythmias, linked to immature hiPSC‐CM electrophysiology, have been reported already in large animal models [66].

Transplantation challenges are exacerbated in DMD patients, where extensive loss of muscle mass, particularly in the heart, compromises the myocardial structure and function, hindering transplanted cardiomyocyte integration and survival. Damaged tissue and inflammation raise the risk of rejection and arrhythmias, while disease progression further impairs this environment, reducing its capacity to support cell engraftment and maturation. Although advanced strategies such as 2D sheets, patches and 3D cardiac models aim to improve engraftment and functional recovery, their application in DMD‐specific transplantation remains largely unexplored, and clinical translation for DMD‐related cardiac dysfunction remains both challenging and speculative.

## 6 Translational Challenges of DMD Therapies

Although diverse therapeutic strategies for DMD are under investigation, clinical translation remains hindered by significant biological, technical and regulatory obstacles (Figure S1). Phenotypic heterogeneity and advanced disease at diagnosis further complicate trial design and patient selection [116].

The large size of the dystrophin gene exceeds standard viral vector capacity, requiring the use of truncated forms such as microdystrophin or dual‐vector intein systems to reconstitute the full‐length protein. However, these approaches remain technically complex with unknown long‐term safety and efficacy. Immune response to vectors and dystrophin pose further barriers, especially in patients with null mutations, who may mount a stronger immune reaction to a previously unseen protein. Manufacturing challenges include high costs, batch variability and low scalability, particularly in the production of AAV vectors and the development of hiPSC‐derived therapies [117, 118].

Gene editing raises concerns about genomic stability and safety, while cell therapies face immunogenicity and tumorigenicity issues [119]. Delivery to the heart remains a critical challenge due to systemic inefficiency, with high doses raising toxicity and immune response risks [120]. Although hiPSC‐CM therapies hold potential, they remain in early experimental stages and face engraftment, maturation and arrhythmia challenges. Moreover, direct therapeutic application of 3D cardiac systems remains speculative. Bridging experimental success and clinical utility requires technical innovation and delivery strategies tailored to cardiac challenges in DMD.

## 7 Conclusions

This update highlights recent advances and ongoing challenges in DMD treatments, emphasizing the complexity of addressing both skeletal and cardiac muscle degeneration. Although FDA‐approved therapies like RNA‐based approaches and microdystrophin gene therapy mark progress, robust evidence supporting their long‐term efficacy remains limited. Gene therapies face challenges due to dystrophin's large gene size and mutation variability, but minidystrophins/microdystrophins and split intein strategies offer potential solutions. Partial dystrophin restoration (~50%) may improve cardiac function in preclinical models, yet data from female carriers reveal that mosaic dystrophin expression can still result in significant cardiac abnormalities. This underscores that high dystrophin‐positive cell proportions are crucial to minimize dysfunction and arrhythmia risk. Effective cardiac targeting remains a bottleneck, with high vector doses raising immune and toxicity concerns, shifting focus towards heart‐specific delivery. Cell therapies, including mesoangioblasts and hiPSC‐derived cells, show promise but face substantial obstacles, including poor cell survival, immune rejection and targeting critical muscles such as the heart and diaphragm, alongside arrhythmia risk.

Recent studies using 3D EHTs/COs from DMD patient–derived hiPSCs have recapitulated key aspects of cardiac dysfunction, such as contractile deficits, calcium dysregulation, fibrosis and oxidative stress, offering mechanistic insights and quantifiable endpoints for therapeutic screening. Although 3D cardiac models provide powerful in vitro platforms for disease modelling and therapy testing, their direct clinical application in patients remains speculative due to challenges in vascularization, integration and long‐term functionality in vivo.

Overall, progress in DMD treatment will depend on combining genetic therapies with approaches addressing pathophysiology, improved cardiac delivery and personalized designs. Future integrative strategies may combine gene editing, patient‐specific 3D models and next‐generation delivery systems, promising more precise and effective treatments for both skeletal and cardiac muscle involvement in DMD.

## Conflicts of Interest

The authors declare no conflicts of interest.

## Supporting information


**Figure S1:** Translational roadmap for cell‐based therapies in DMD. The schematic illustrates general examples of how cell‐based strategies move from preclinical studies using muscle and hiPSC‐derived cells, CRISPR/Cas9 gene correction and animal models, through key translational challenges such as poor engraftment, cardiac delivery barriers, immunogenicity and systemic administration, towards early clinical testing. Ongoing Phase 1/2 and 3 trials include iPSC‐derived progenitors, HLA‐matched mesoangioblasts and cardiosphere‐derived cells (CAP‐1002), while DMD‐specific transplantation approaches remain speculative.

**Table S1:** Comparison of current gene therapy strategies in DMD. Main genetic strategies used in preclinical and clinical trials. AAV (Myo)—(muscle‐tropic) adeno‐associated vector, CPP—cell‐penetrating peptide, PMOs—phosphorodiamidate morpholino oligomers, PPMOs—peptide‐conjugated phosphorodiamidate morpholino oligomers, rAAVrh74—recombinant AAV serotype rh74, scAAV—self‐complementary AAV.
