# Supplementary figures and images for: Current Trends in Duchenne Muscular Dystrophy Research and Therapy: 3D Cardiac Modelling
Source: J Cachexia Sarcopenia Muscle. 2026 Jan 7;17(1):e70180. doi: 10.1002/jcsm.70180 (PMC12776602; doi:10.1002/jcsm.70180)

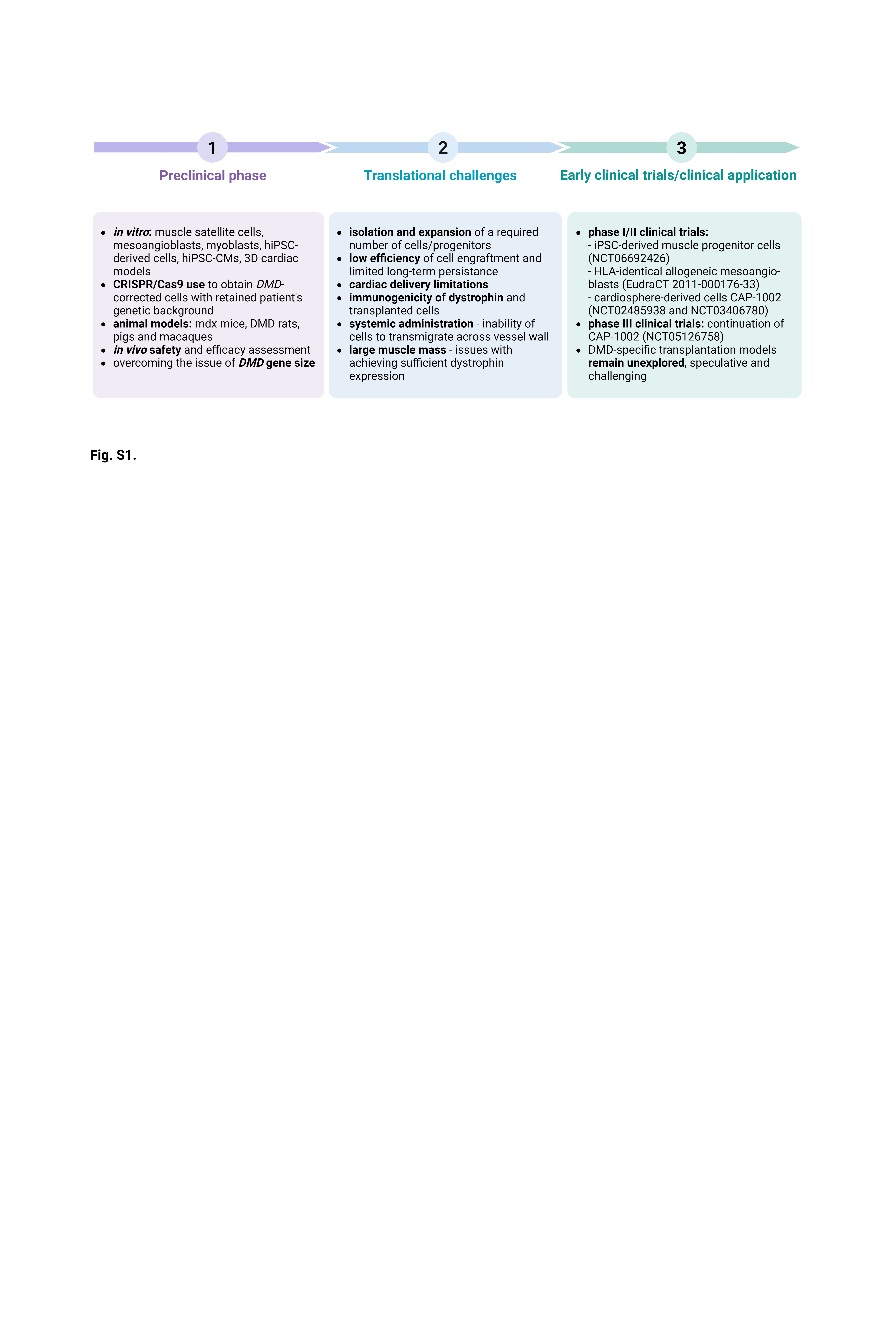

Supplement: Supplementary file 1 — Figure S1: Translational roadmap for cell‐based therapies in DMD. The schematic illustrates general examples of how cell‐based strategies move from preclinical studies using muscle and hiPSC‐derived cells, CRISPR/Cas9 gene correction and animal models, through key translational challenges such as poor engraftment, cardiac delivery barriers, immunogenicity and systemic administration, towards early clinical testing. Ongoing Phase 1/2 and 3 trials include iPSC‐derived progenitors, HLA‐matched mesoangioblasts and cardiosphere‐derived cells (CAP‐1002), while DMD‐specific transplantation approaches remain speculative. [file JCSM-17-e70180-s002.jpg]
