# Supplementary material for: Current Trends in Duchenne Muscular Dystrophy Research and Therapy: 3D Cardiac Modelling
Source: J Cachexia Sarcopenia Muscle. 2026 Jan 7;17(1):e70180. doi: 10.1002/jcsm.70180 (PMC12776602; doi:10.1002/jcsm.70180)
Supplement: Supplementary file 2 — Table S1: Comparison of current gene therapy strategies in DMD. Main genetic strategies used in preclinical and clinical trials. AAV (Myo)—(muscle‐tropic) adeno‐associated vector, CPP—cell‐penetrating peptide, PMOs—phosphorodiamidate morpholino oligomers, PPMOs—peptide‐conjugated phosphorodiamidate morpholino oligomers, rAAVrh74—recombinant AAV serotype rh74, scAAV—self‐complementary AAV. [file JCSM-17-e70180-s001.docx]

**Table S1. Comparison of current gene therapy strategies in DMD.** Main genetic strategies used in preclinical and clinical trials. AAV(Myo) – (muscle-tropic) adeno associated vector, CPP – cell-penetrating peptide, PMOs - phosphorodiamidate morpholino oligomers, PPMOs – peptide-conjugated phosphorodiamidate morpholino oligomers, rAAVrh74 – recombinant AAV serotype rh74, scAAV – self-complementary AAV.

| Genetic strategy | | Type of modulation | Mechanism of action | Key features | Advantages | Limitations |
| --- | --- | --- | --- | --- | --- | --- |
| **PMOs**  FDA approved variants: Eteplirsen, Golodirsen, Vitolarsen, Casimersen | | mRNA modulation | exon skipping (splicing modulation) | synthetic antisense oligonucleotides targeting pre-mRNA splicing | **✓** high binding specificity **✓** low immunogenicity  **✓** FDA approved variants | **X** require repeated dosing **X** mutation-specific  **X** poor cardiac delivery  **X** various % of dystrophin expression  **X** adverse effects occurrence |
| **PPMOs** | | mRNA modulation | exon skipping  (splicing modulation) | enhanced cell uptake via CPP (cell-penetrating peptide) conjugated to  the PMO backbone in skeletal and cardiac cells | **✓** high binding specificity  **✓** improved cellular uptake in skeletal and cardiac muscle cells  **✓** better distribution to skeletal and cardiac muscle | **X** require repeated dosing  **X** mutation-specific  **X** potentially increased immunogenicity (peptide-dependent)  **X** potential toxicity  **X** adverse effects occurrence **X** in preclinical/early clinical stages |
| **AAV- based** | SCAAV9.U7.ACCA | mRNA modulation | exon skipping  (splicing modulation) | self-complementary AAV9 encoding U7 snRNA for long-term splicing modulation | **✓** natural tropism to skeletal muscle and heart  **✓** expression in skeletal and cardiac muscle  **✓** systemic single dose  **✓** long-term splicing modulation | **X** immunogenicity related to AAV vector **X** limited to certain mutations **X** in preclinical/early clinical stages |
|  | rAAVrh74  FDA approved variant: Elevidys | DNA delivery | AAV-mediated delivery of truncated *DMD* gene | uses MHCK7 promoter for targeted muscle expression | **✓** natural tropism to skeletal muscle and heart  **✓** muscle-specific promoter (MHCK7) **✓** expression in skeletal and cardiac muscle  **✓** systemic single dose  **✓** FDA approved variant | **X** only truncated dystrophin restoration  **X** potential immunogenicity related to AAV vector and truncated dystrophin **X** dose-dependent safety concerns  **X** Elevidys - paused June 15, 2025; resumed July 28, 2025, for ambulatory patients; non-ambulatory use still on hold |
|  | AAVMyo | DNA delivery | AAV-mediated delivery of truncated/full-length *DMD* gene | allows trans-splicing mediated by split inteins  enhanced muscle tropism | **✓** enhanced tropism to skeletal and cardiac muscle  **✓** engineered for high efficiency in skeletal and cardiac muscle  **✓** allows truncated/full-length dystrophin expression with use of split inteins  **✓** systemic single dose  **✓** lower systemic exposure (targets muscle tissue)  **✓** lower doses | **X** immunogenicity related to AAV vector and dystrophin expression  **X** in preclinical phase |
| **CRISPR-based** | | DNA modulation | genome editing (exon deletion/  correction) | direct, potentially permanent gene correction at DNA level | **✓** curative potential **✓** systemic single dose **✓** multi-exon strategies possible | **X** delivery efficiency  **X** high AAV doses to deliver system components  **X** immunogenicity related to AAV vector, Cas9 protein, and newly expressed dystrophin  **X** off-target risk **X** in preclinical/early clinical stages |
